# Knowledge, perception and attitude toward fibromyalgia among physical therapists in the United Arab Emirates: A cross-sectional study

**DOI:** 10.1371/journal.pone.0341454

**Published:** 2026-02-04

**Authors:** Mona A. Almulla, Amna M. Farhani, Emad A. Aboelnasr, Fatma A. Hegazy

**Affiliations:** 1 Department of Physiotherapy, College of Health Sciences, University of Sharjah, Sharjah, United Arab Emirates; 2 Faculty of Physical Therapy, Cairo University, Cairo, Egypt; Prime Hospital LLC, UNITED ARAB EMIRATES

## Abstract

**Background:**

Fibromyalgia (FM) is a chronic condition classified by widespread pain, fatigue, and associated symptoms. Patients with FM are frequently referred to physical therapists, whose knowledge of assessment criteria and management strategies is critical for timely recognition and effective care. Early diagnosis has been shown to improve outcomes, whereas delayed recognition often leads to prolonged suffering and increased healthcare costs.

**Aim:**

The objective of this study was to examine the knowledge, perceptions, and attitudes of physical therapists in the United Arab Emirates (UAE) with respect to the diagnosis and management of FM.

**Methods:**

A cross-sectional self-reported survey was distributed electronically to practicing physical therapists across the UAE. The survey collected demographic data, as well as information on confidence in determining and managing FM, awareness of international guidelines, perceptions of other healthcare providers roles, and knowledge of the risk factors.

**Results:**

A total of 300 physical therapists were invited, and 240 completed the survey and met the inclusion criteria (response rate of 80%). The results revealed a predominantly female workforce, with 73.8% of participants identifying as female. The age of most respondents ranged between 23 and 42 years. Almost half of the participants had less than five years of experience. Nearly two-thirds of participants expressed confidence in diagnosing and managing FM cases. Most participants were unaware of any of the international FM practice guidelines (1990 ACR, 2010 ACR, 2012 Canadian).

**Conclusion:**

The findings of this study underscore a concern for a lack of confidence and awareness among physical therapists in the UAE regarding the diagnosis and management of FM cases. Despite a significant proportion of participants reporting experience in managing FM cases, the majority were not familiar with recent FM practice guidelines, indicating potential gaps in knowledge and practice. This study highlights the importance of improving curricular integration of FM content, and greater dissemination of evidence-based guidelines. Addressing these gaps will be essential for promoting earlier diagnosis, reducing delays in management, and improving patient outcomes in the UAE.

## Introduction

Fibromyalgia syndrome (FM) is a chronic condition classified by widespread pain, joint stiffness, and tenderness of the muscles and tendons [1]. Several additional symptoms, such as fatigue, cognitive impairment, sleep resistance, and psychosocial difficulties, commonly accompany the disorder, further complicating its clinical picture [2,3]. Although the exact etiology of FM remains uncertain, recent research has provided valuable insights into its multifactorial pathophysiology [4].

FM is one of the most impairing causes of chronic musculoskeletal pain, with an estimated global prevalence of 2%–6%. The condition disproportionately affects women, particularly between the ages of 30–55 years, and prevalence can be as high as 7% among women aged 50–80 years, with a female-to-male ratio of approximately 3:1 [5,6]. Diagnostic approaches have evolved over time: the American College of Rheumatology (ACR) introduced the term “fibromyalgia syndrome” in the 1990s [7], and subsequent revisions in 2010 and 2011 incorporated the Symptom Severity Scale (SSS) and the Widespread Pain Index (WPI) into updated criteria [8]. These refinements aimed to improve diagnostic reliability; however, diagnosis remains challenging because patients often present with vague or overlapping complaints, leading to delayed or missed recognition. Delayed diagnosis not only worsens patient outcomes but also increases the overall burden of care [9].

A consistent barrier in FM management is limited professional awareness of the diagnostic range and evidence-based treatment guidelines [10]. Previous studies among physicians and rheumatologists have highlighted this knowledge gap, with nearly half of surveyed clinicians unfamiliar with the ACR criteria [11,12]. Other studies have revealed significant variations in perceptions and knowledge of diagnosing and treating FM, not only between different specialties but also within the same specialty [13]. Studies generally reported high percentages of participants with a poor knowledge about FM diagnosis and management [12]. Such deficits contribute to inconsistent care, unnecessary investigations, higher costs, and decrease quality of life for patients [13].

Within the multidisciplinary management of FM, physical therapists (PTs) play a significant role, as they provide non-pharmacological interventions including patient education, structured exercise programs, and pain management modalities [14,15]. However, international evidence indicates that physical therapists themselves often report uncertainty or lack of confidence when diagnosing and managing FM. For instance, a study in Saudi Arabia revealed significant gaps in physical therapists’ awareness of FM diagnostic guidelines and management practices [16], while similar concerns have been echoed in other contexts [17]. These studies emphasize the necessity for comprehensive, evidence-based educational programs to address these gaps in knowledge and awareness among physical therapists.

Despite the growing burden of FM, no published data currently exist on the awareness, perceptions, and attitudes of physical therapists toward FM diagnosis and management in the United Arab Emirates (UAE). Moreover, the UAE context is unique: its healthcare system is rapidly expanding, its PT workforce is highly multicultural, and there are limited formal continuing education programs addressing FM specifically. Additionally, there are no reliable prevalence estimates of FM in the UAE.

Consequently, the objective of this study is to determine the knowledge, perceptions, and attitudes of physical therapists in the UAE with respect to FM assessment and management. The study aims to inform targeted training initiatives and contribute to the integration of evidence-based practice within the UAE’s physical therapy profession by addressing this disparity.

## Materials and methods

### Ethics statement

This study received the approval from university of Sharjah ethical review committee no, (REC-22-10-02-S).

Participants were provided with information describing the nature of the study, study purpose and explaining that no foreseeable risks existed for participation and their right to withdraw from the research study on their own free well. Additionally, the first page of the survey included the informed consent text and required the participants to acknowledge the terms prior to commencing the survey questions by ticking a check box meaning written consent form was obtained prior to participating in this study. The online survey system was hosted in Google Survey. No one other than the researcher has access to the administrative interface of the website. Furthermore, the results of the survey were kept confidential and only shared with those involved. In summary, several preventive and declarative steps ensured that the research conducted in an ethical manner.

### Study design

To examine the knowledge, perceptions, and attitudes of physical therapists in the UAE with respect to FM diagnosis and management, a descriptive cross-sectional design was implemented.

### Participants and sampling

Physical therapists currently practicing in public and private healthcare sectors across the UAE were eligible to participate. Physical therapy students and interns were excluded because of their limited clinical exposure to FM management. A total of 300 licensed physical therapists were invited to participate through electronic invitations disseminated via professional networks and social media platforms. Of these, 240 therapists completed the survey, yielding a response rate of 80%.

A convenience sampling technique was employed due to the absence of a centralized registry or institutional list of practicing physical therapists in the UAE. The minimum required sample size (n = 234) was evaluated using G*Power 3.1.9.4 software, with parameters set at α = 0.05, power (1-β) = 0.80, and effect size w = 0.20. Thus, the final sample of 240 participants satisfied the calculated requirement.

### Study survey

The self-administered survey was adapted, with permission, from the validated questionnaire developed by Alodiabi et al. (2020) [16]. It was organized into three major sections:

1. **Informed consent and study overview** – detailing the study purpose, eligibility criteria, anonymity assurances, and consent checkbox.
2. **Demographic and professional information** – including gender, age, nationality, educational qualifications, years of practice, and Emirate of practice.
3. **FM-related questions** – including:

Questions that evaluated the participant’s educational background and sources of information about FM, their level of confidence in assessing FM (5-point Likert type scale), confidence in managing FM (5-point Likert type scale), knowledge and awareness of common FM diagnostic criteria and management guidelines (1990 ACR [18], 2010 ACR [8], 2012 Canadian Guidelines [19]), perceptions of different healthcare professions and their roles in managing FM patients, knowledge and awareness of FM risk factors, the level of compliance with common recommendations for FM interventions (4-point Likert scale type), and a quiz on general knowledge of FM (true/false questions).

### Statistical analysis

Means with standard deviations were employed to summarise continuous variables, while frequencies and percentages were employed for categorical variables. The Chi-square test was employed to analyse group comparisons, and Spearman’s rank correlation coefficient was employed to investigate correlations among variables. Statistical significance was examined by p-values less than 0.05. IBM SPSS (Version 22.0) was used to perform all analyses.

## Results

A total of 300 licensed physical therapists were invited to participate in the survey, of whom 240 responded and completed the questionnaire, yielding a response rate of 80%. The sample represented a wide age range of professionals practicing in the UAE. Our findings revealed a predominantly female workforce, with 73.8% of participants identifying as female. Most participants were non-Emirati nationals comprising 90.4%, While Emirati participants accounted for only 9.6%.

Regarding educational achievement, 36.7% of participants had postgraduate credentials, such as master’s and doctoral degrees, while 63.3% had a graduate degree, or bachelor’s degree. Most professionals (73.3%) earned their highest degree after 2010. Less than five years of clinical expertise was possessed by nearly half of the participants. The study encompassed physical therapists from different Emirates within the UAE, with the largest proportion practicing in Dubai (41.7%). The study sample’s demographic and professional attributes (N = 240) are shown in Table 1.

**Table 1:** The study sample’s professional and demographic attributes (N = 240).

Variable	Frequency	%
Gender		
Males	63	26.3
Females	177	73.8
		
Age		
<23y	10	4.2
23-32y	131	54.6
33-42y	68	28.3
43-52y	25	10.4
>52y	6	2.5
Nationality		
Emirati	23	9.6
Non-Emirati	217	90.4
Highest educational degree		
Graduate	152	63.3
Postgraduate	88	36.7
Year of highest educational degree		
1980–1990	3	1.3
1991–2000	11	4.6
2001–2010	50	20.8
After 2010	176	73.3
Years of experience (Years)		
< 2	58	24.2
2-5	57	23.8
6-10	49	20.4
11-15	35	14.6
16-20	20	8.3
> 20	21	8.8
Emirates of practice		
Abu Dhabi	46	19.2
Ajman	27	11.3
Dubai	100	41.7
Fujairah	4	1.7
Ras Al-Khaimah	5	2.1
Sharjah	54	22.5
Umm Al-Quwain	4	1.7

### Participants’ confidence in assessing, diagnosing, and managing fibromyalgia (FM) Cases

This study examined the confidence levels of physical therapists in the assessment, diagnosis, and management of FM cases. On a five-point Likert scale, participants were requested to designate their level of confidence, with response options ranging from “not confident” to “extremely confident.”

**Fig 1:**
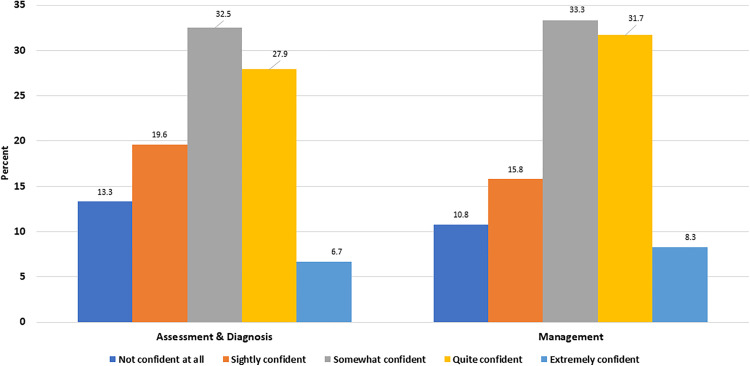
Participants’ confidence levels in assessing, diagnosing, and managing FM cases.

A total of 240 physical therapists from various healthcare settings participated in the survey. The results revealed interesting insights into the participants’ confidence levels in different aspects of Fibromyalgia care (Fig 1).

Nearly two-thirds of participants expressed confidence in their ability to assess and diagnose FM cases, with (32.5%) reporting being somewhat confident, (27.9%) quite confident, or (6.7%) extremely confident. However, a notable proportion (13.3%) indicated no confidence at all in this aspect of care. When it came to managing FM cases, participants demonstrated varying levels of confidence, with (65%) reporting being somewhat to quite confident. However, a noteworthy proportion (15.8%) expressed lower confidence levels, and (10.8%) showed no confidence at all in managing this condition.

### Knowledge and Awareness of Common FM Diagnostic Criteria and Management Guidelines

**Fig 2:**
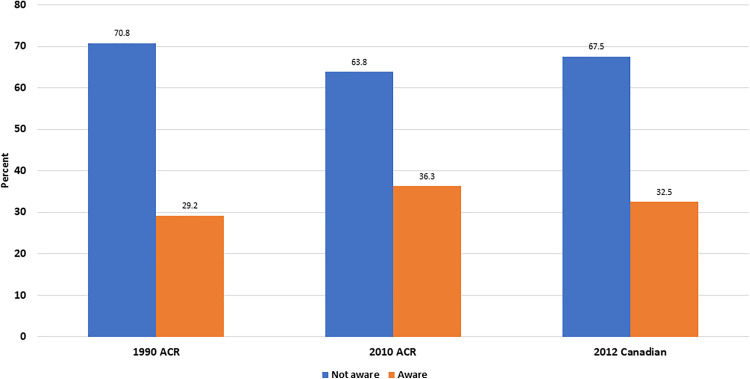
Participants’ knowledge and awareness of three common FM practice guidelines.

We evaluated the information and cognisance of participants with respect to three prevalent FM practice guidelines. The participants were requested to specify whether they were aware of each of the practice guidelines (1990 ACR [18], 2010 ACR [8], 2012 Canadian Guidelines [19]). The findings showed that none of the three FM practice guidelines were known to the majority of participants (Fig 2).

### Perceptions of the Roles of Diverse Healthcare Professions in the Management of Fibromyalgia Cases

This study examined participants’ perspectives regarding the roles of various healthcare providers in the management of FM. Participants were asked to indicate whether they believed each type of healthcare provider had no role, a primary role, a secondary role, or if they did not know the role of each provider in managing FM.

The majority of participants believed that physical therapists and pain management physicians played a primary role in the management of FM, with 76.2% and 72.9% representing the respective percentages. However, one-quarter of participants (25%) saw physical therapists in a secondary role.

Participants had varying perceptions of the role of rheumatologists in FM management. While 56.3% considered rheumatologists to have a primary role, a large proportion (28.3%) saw them in a secondary role. About 6.7% believed that rheumatologists had no role in FM management, and 8% were unsure of their role.

**Fig 3:**
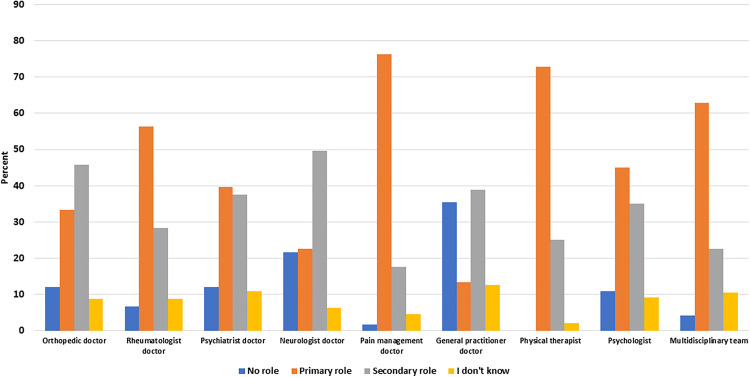
Participants’ perceived roles of each type of healthcare provider in managing FM.

In the case of psychologists/psychiatrists, 39.6% of participants considered them to have a primary role in FM management, while a similar proportion, 37.5%, saw them in a secondary role. Only 12.1% believed that psychologists/psychiatrists had no role in FM management, and 10.8% were unsure of their role (Fig 3).

### Participants’ Understanding and Awareness of FM Risk Factors

The knowledge and awareness of risk factors associated with FM of the participants were also investigated in this study. Participants were presented with a list of potential risk factors and asked to categorize each as either “not a risk factor,” “a risk factor,” or “I don’t know.”

The results indicated varying levels of knowledge and awareness among participants regarding the potential risk factors for FM. About two-thirds of participants (57.9%) correctly recognized genetic factors as a risk factor for FM. However, a notable proportion (22.5%) expressed uncertainty about this factor, and (19.6%) incorrectly considered it as not a risk factor. In contrast, most participants (79.6%) correctly identified poor sleep patterns as a risk factor for FM. Nevertheless, (12.9%) were unsure about this factor, and (7.5%) considered it as not a risk factor.

**Fig 4:**
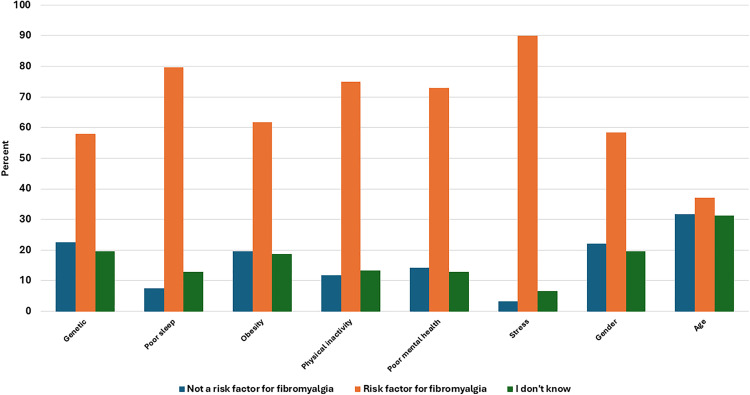
Participants’ knowledge and awareness of risk factors for FM.

Participants demonstrated a reasonable level of awareness regarding the role of physical inactivity and poor mental health on FM, with (75%) identifying physical inactivity and (72.9%) identifying poor mental health as risk factors for this condition. However, participants seem to agree regarding the role of Stress, as a significant majority (90%) recognized it as a risk factor for FM (Fig 4).

### Comparative analysis

The results of our study, which compared the knowledge of clinical practice guidelines for managing FM among undergraduate and postgraduate physical therapists, are presented in this section.

A series of queries regarding the recommended management strategies and interventions were administered to participants in order to evaluate their understanding of clinical practice guidelines for managing FM. The association between the level of education (undergraduate vs. postgraduate) and knowledge level was investigated using a Chi-Square analysis.

Graduates and postgraduates’ educational attainment and familiarity with the 1990 ACR Guidelines were found to be statistically significantly correlated by the Chi-Square analysis (χ² = 11.16, df = 1, p = 0.001). This result suggests that the two groups’ knowledge levels differ significantly from one another. Specifically, postgraduates demonstrated a higher level of knowledge regarding the 1990 ACR Guidelines in comparison to graduates. (See Table 2)

**Table 2:** The comparative analysis of participants’ knowledge of the 1990 ACR.

Crosstab					
			Highest education		Total
			Grad	Post	
1990 ACR	No	Count	119	51	170
		% within Highest education	78.3%	58.0%	70.8%
	Yes	Count	33	37	70
		% within Highest education	21.7%	42.0%	29.2%
Total		Count	152	88	240
		% within Highest education	100.0%	100.0%	100.0%
					
Chi-Square Tests					
	Value	Df	p-value	p-value	p-value
χ²	11.155(b)	1	0.001		
Continuity Correction(a)	10.193	1	0.001		
Likelihood Ratio	10.930	1	0.001		
Fisher’s Exact Test				0.001	0.001
N	240				

a. Computed only for a 2x2 table.

b. 0 cells (.0%) have an anticipated count of less than 5. The minimum predicted count is 25.67.

Similarly, the results of our comparative analysis between graduates and postgraduates regarding their knowledge of the 2010 ACR Guidelines revealed a statistically significant association (χ² = 13.32, df = 1, p < 0.001). This significant finding underscores a marked disparity in knowledge levels between the two groups. Specifically, postgraduates exhibited a notably higher level of familiarity and understanding of the 2010 ACR Guidelines compared to graduates. (See Table 3)

**Table 3:** The comparative analysis of participants’ knowledge of the 2010 ACR.

Crosstab					
			Highest education		Total
			Grad	Post	
2010 ACR	No	Count	110	43	153
		% within Highest education	72.4%	48.9%	63.8%
	Yes	Count	42	45	87
		% within Highest education	27.6%	51.1%	36.3%
Total		Count	152	88	240
		% within Highest education	100.0%	100.0%	100.0%
					
Chi-Square Tests					
	Value	Df	p	p	p
χ²	13.324(b)	1	0.000		
Continuity Correction(a)	12.326	1	0.000		
Likelihood Ratio	13.186	1	0.000		
Fisher’s Exact Test				0.000	0.000
N	240				

a. Computed only for a 2x2 table.

b. 0 cells (.0%) have expected count less than 5. The minimum expected count is 31.90.

However, the results of the comparative analysis between graduates and postgraduates regarding their knowledge of the 2012 Canadian Guidelines did not yield a statistically significant association (χ² = 0.95, df = 1, p = 0.33). This finding indicates that there is no substantial difference in knowledge levels between the two groups with respect to the 2012 Canadian Guidelines. Both graduates and postgraduates demonstrated similar levels of familiarity and understanding of these particular clinical guidelines. (See Table 4).

**Table 4:** The comparative analysis of participants’ knowledge of the 2012 Canadian Guidelines.

Crosstab					
			Highest education		Total
			Grad	Post	
2012 Canadian	No	Count	106	56	162
		% within Highest education	69.7%	63.6%	67.5%
	Yes	Count	46	32	78
		% within Highest education	30.3%	36.4%	32.5%
Total		Count	152	88	240
		% within Highest education	100.0%	100.0%	100.0%
					
Chi-Square Tests					
	Value	Df	p-value	p-value	p-value
χ²	.945(b)	1	0.331		
Continuity Correction(a)	0.688	1	0.407		
Likelihood Ratio	0.939	1	0.333		
Fisher’s Exact Test				0.391	0.203
N	240				

a. Computed only for a 2x2 table.

b. 0 cells (.0%) have an anticipated count of less than 5. The minimum predicted count is 28.60.

### Correlational analysis

In this section, we present the observations of the correlational analysis, a critical component of our study that examines the relationships between participants’ years of experience and several variables of interest. Correlational analysis allows to explore the degree and direction of associations between different factors without inferring causation. These rigorous statistical techniques aim to uncover meaningful patterns and connections within our data, shedding light on the potential interplay between the variables under investigation.

The correlational analysis between participants’ years of experience and their knowledge of the 1990 ACR, the 2010 ACR, and the 2012 Canadian guidelines revealed statistically significant associations (See Table 5). Specifically, there was a positive statistical association between participants’ years of experience and their knowledge of the 1990 ACR guidelines (p = 0.002), indicating that as participants’ years of experience increased, their familiarity with the 1990 ACR guidelines also tended to rise.

**Table 5:** The correlational analysis results.

Correlations			
			Years of experience
Spearman’s rho	1990 ACR	Ρ	0.202
		p-value	**0.002**
		N	240
	2010 ACR	Ρ	0.219
		p-value	**0.001**
		N	240
	2012 Canadian	Ρ	0.143
		p-value	**0.026**
		N	240
	Pain education	Ρ	0.026
		p-value	0.685
		N	240
	Self-management	Ρ	−0.039
		p-value	0.547
		N	240
	Aerobic exercises	Ρ	0.108
		p-value	0.095
		N	240
	Cognitive behavioural therapy (CPT)	Ρ	0.169
		p-value	**0.009**
		N	240
	Manual therapy	Ρ	0.020
		p-value	0.756
		N	240
	Dry needling	Ρ	0.082
		p-value	0.205
		N	240
	Ultrasound/electrotherapy	Ρ	−0.013
		p-value	0.840
		N	240
	Massage	Ρ	0.041
		p-value	0.531
		N	240
	Acupuncture	Ρ	0.057
		p-value	0.376
		N	240

Similarly, a positive statistical association was observed between years of experience and knowledge of the 2010 ACR Guidelines (p = 0.001), suggesting that individuals with more years of experience were more likely to have a better understanding of the 2010 ACR Guidelines.

Furthermore, the analysis revealed a positive statistical association between years of experience and knowledge of the 2012 Canadian guidelines, although the association was comparatively weaker (p = 0.026). This finding suggests that participants with greater years of experience tended to have a somewhat higher level of familiarity with the 2012 Canadian guidelines.

Additionally, a statistically significant positive correlation was observed between years of experience and knowledge of Cognitive Behavioral Therapy (CBT) (p = 0.009). This suggests that as participants’ years of professional experience increased, their awareness and understanding of CBT as an intervention for managing fibromyalgia cases tended to be higher.

However, it is noteworthy that no statistically significant associations were found between years of experience and familiarity with a range of other interventions commonly used to treat fibromyalgia, including massage therapy, acupuncture, ultrasound/electrotherapy, manual therapy, and dry needling (Table 5). These results indicate that years of professional experience did not appear to significantly influence participants’ familiarity with these specific interventions for managing fibromyalgia.

## Discussion

Fibromyalgia is a complex and often misunderstood condition that poses diagnostic and management challenges for healthcare practitioners generally and especially for physical therapists, since they deal primarily with pain management [20]. Examining the knowledge and awareness of physical therapists working in the United Arab Emirates regarding the diagnosis and treatment of fibromyalgia was the aim of this study. The results offer valuable insights into the present state of knowledge and potential areas for improvement within the physical therapy profession in the UAE.

The descriptive analysis of participants’ demographic and professional characteristics highlights a diverse, well-educated, and experienced physical therapy workforce, reflecting the multicultural composition of healthcare providers in the UAE. While a substantial portion of physical therapists reported confidence in assessing, diagnosing, and managing FM cases, a significant number lacked adequate confidence and awareness in these areas. Such challenges are consistent with prior reports. For instance, Alodiabi et al [16]. documented limited awareness and confidence among physical therapists in Saudi Arabia. Similarly, studies from Pakistan and Canada have shown that both physicians and therapists often demonstrate inadequate familiarity with FM diagnostic criteria and guidelines [21,22]. However, in Western countries such as the US and parts of Europe, surveys have indicated relatively higher awareness levels, which may reflect stronger integration of FM content in professional education and continuing medical training [12]. These international differences suggest that structural factors—such as curriculum content, national guidelines, and cultural attitudes toward chronic pain—may explain variations in professional preparedness.

One critical implication of this knowledge gap is its potential impact on the timeliness of diagnosis. Previous research has shown that early recognition of FM is associated with better management outcomes, reduced symptom burden, and improved patient quality of life, whereas delayed diagnosis often results in prolonged suffering, higher healthcare costs, and increased risk of comorbidities [22]. In the UAE context, the finding that many physical therapists lack familiarity with established diagnostic guidelines raises concern that patients may face delayed recognition of FM, limiting the effectiveness of interventions and rehabilitation. These outcomes highlight the potential need for targeted training and education programs to enhance clinicians’ confidence, awareness, and competence in providing optimal care for patients with Fibromyalgia. Further research and interventions may be necessary to address these disparities in confidence levels and improve the quality of care for individuals with Fibromyalgia.

The results also demonstrate that participants had diverse perceptions of the roles of different healthcare providers in managing FM. While many viewed physical therapists and pain physicians as having a primary role, a notable proportion showed uncertainty about the responsibilities of rheumatologists and psychologists. This mirrors findings from Spain and Canada, where confusion about interdisciplinary roles in FM care has been reported [23,24]. Such ambiguity may undermine team-based care and negatively affect patient experiences. Clearer communication, role definition, and interprofessional collaboration are therefore essential to ensure holistic management.

Our comparative analysis revealed that postgraduate-trained therapists demonstrated greater knowledge of FM guidelines compared to graduates, aligning with findings from Canada by Kumbhare et al. [25], who noted that clinicians with advanced training were more likely to adhere to diagnostic criteria. These results underscore the importance of advanced education and specialized training in bridging knowledge gaps. The correlational analysis further highlighted the role of clinical experience, as therapists with more years of practice were more familiar with established guidelines and evidence-based interventions such as cognitive-behavioral therapy (CBT). However, years of experience did not appear to significantly influence knowledge of other interventions, suggesting that ongoing professional development is necessary to maintain a broad and up-to-date skill set.

Overall, these findings point to an urgent need for targeted continuing education programs and structured workshops in the UAE, focusing on FM diagnostic criteria, evidence-based guidelines, and the interdisciplinary nature of FM management. Addressing these gaps may not only enhance therapist confidence but also promote earlier diagnosis and better outcomes for patients.

### Limitations

When evaluating the results, it is important to take into account the many limitations of this study. First, because younger or more technologically savvy therapists were more likely to engage, selection bias may have been introduced by the use of convenience sampling through internet and social media platforms. The demographic profile shows that most of the responders were under 40, which reflects this. Second, the absence of an official national registry of licensed physical therapists in the UAE limited our ability to establish a precise sampling frame. To our knowledge, there is no publicly available official registry of licensed PTs in the UAE; thus, we could not provide exact population figures. As a result, while the sample size met statistical requirements, the extent to which it represents the broader population of UAE physical therapists cannot be fully determined.

Third, the use of self-reported data may have caused participants to exaggerate their knowledge or confidence, which raises the possibility of social desirability bias and recollection bias. Fourth, the cross-sectional design precludes any conclusions about causality or changes in knowledge and perceptions over time. Finally, although the survey was adapted from a validated instrument, cultural and contextual differences may have influenced how participants interpreted some items.

In order to overcome these constraints, future studies should use probability-based sampling techniques, integrating data from professional licensing bodies to ensure representativeness, and adopting longitudinal or mixed-methods designs to capture changes in knowledge and practice over time.

## Conclusion

This study offers the first understanding of the attitudes, beliefs, and knowledge of physical therapists in the United Arab Emirates with regard to fibromyalgia. The majority of therapists were not familiar with accepted international diagnostic criteria and management recommendations, even though many expressed some level of confidence in their ability to diagnose and treat FM. This highlights a critical gap between clinical exposure and evidence-based knowledge. Postgraduate education and greater years of experience were associated with higher awareness of guidelines, suggesting that advanced training contributes meaningfully to preparedness. However, the overall findings indicate that misconceptions and uncertainty remain widespread.

These results emphasize the urgent need for targeted continuing professional development programs, integration of FM content into physical therapy curricula, and wider dissemination of practice guidelines. Strengthening the knowledge base of UAE physical therapists is particularly important to support earlier recognition of FM, avoid delays in diagnosis, and ensure optimal patient management. The UAE healthcare system can raise the standard of care for people with FM and bring practices into compliance with international norms by filling in these gaps.

## Supporting information

S1 File Questionnaire. (PDF)
