# Supplementary material for: Knowledge, perception and attitude toward fibromyalgia among physical therapists in the United Arab Emirates: A cross-sectional study
Source: PLoS One. 2026 Feb 4;21(2):e0341454. doi: 10.1371/journal.pone.0341454 (PMC12871961; doi:10.1371/journal.pone.0341454)
Supplement: S1 File — (PDF) [file pone.0341454.s001.pdf]

Questionnaire

INFORMED CONSENT

It is my pleasure to invite you to participate in this online study. This research study is being conducted by Master student from the University of Sharjah, College of Health Sciences, Department of Physiotherapy, to determine the level of awareness of fibromyalgia assessment and management among physiotherapist working in the United Arab Emirates.

The survey should take approximately 10-15 minutes to complete.

Your participation is completely voluntary. The data will be kept strictly confidential and for scientific research purposes only. You have the right to refuse or to withdraw the survey at any point. If you have questions at any time about the survey or the procedures, you may contact Mona Almulla on +971551663526 or email address: U20105907@sharjah.ac.ae.

If you have concerns or complaints about this study, please write to the University of Sharjah Research Ethics Committee at rec@sharjah.ac.ae.

Thank you for being interested to take part in our survey!

* Required

- I have read the description above and I agree to participate in this research study.

* Mark only one.

☐ Yes
☐ No

Section 1: Demographics

- Age range?

  ☐ < 23 years
  ☐ 23-32 years
  ☐ 33-42 years
  ☐ 43-52 years
  ☐ > 52 years

- What is your gender?
  ☐ Male
  ☐ Female

- Nationality?
  ☐ Emirati
  ☐ Non-Emirati

- Highest level of education
  ☐ BSc
  ☐ MSc
  ☐ DPT
  ☐ PhD
  ☐ Other (specify ….)

- Country from which highest degree achieved
  ☐ UAE
  ☐ US
  ☐ UK
  ☐ Australia
  ☐ other (specify ….)

- Year of graduation with the highest degree?
  ☐ Before 1980
  ☐ 1980 – 1990
  ☐ 1991 – 2000
  ☐ 2001 – 2010
  ☐ After 2010

- Any post-graduate certificates that relate to chronic pain and FM
  ☐ Yes – No
  ☐ If yes, please specify

- Emirates of practice?
  ☐ Sharjah

- ☐ Dubai
- ☐ Abu Dhabi
- ☐ Ajman
- ☐ Ras AlKhaimah
- ☐ Fujairah
- ☐ Umm AlQuwain

- Years of experience
  - ☐ Less than 2 years
  - ☐ 2-5
  - ☐ 6-10
  - ☐ 11-15
  - ☐ 16-20
  - ☐ More than 20 years of experience

The following 9 questions (4.1 to 4.9) aims to study the knowledge, attitude and perception of physical therapist working in the UAE about fibromyalgia (FM):

1.1 where did you receive your knowledge about fibromyalgia? (Select one or more from the following)

- ☐ Undergraduate education
- ☐ Post-graduate education
- ☐ Self-learning
- ☐ I don't have knowledge about fibromyalgia
- ☐ other (specify ….)

1.2 how many FM patients you see per year? (Select one from the following)

- ☐ 0
- ☐ 1 -5
- ☐ 6 – 10
- ☐ 11 – 15
- ☐ More than 15

1.3 how confident are you in your ability to assess and diagnose FM patients?

- ☐ Not confident at all
- ☐ Sightly confident
- ☐ Somewhat confident
- ☐ Quite confident
- ☐ Extremely confident

1.4 How confident are you in your ability manage and treat FM patients?

- ☐ Not confident at all
- ☐ Sightly confident
- ☐ Somewhat confident
- ☐ Quite confident
- ☐ Extremely confident

1.5 Are you aware of each of the following fibromyalgia (FM) diagnostic criteria/ guidelines?

- 1990 ACR (The American college of Rheumatology 1990 criteria)
  - ☐ Yes, I am aware of this
  - ☐ No, I am not aware of this

- 2010 ACR (The American college of Rheumatology 2010 criteria)
  - ☐ Yes, I am aware of this
  - ☐ No, I am not aware of this

- 2012 Canadian Guidelines for the diagnosis and management of fibromyalgia
  - ☐ Yes, I am aware of this
  - ☐ No, I am not aware of this
- Aware of other FM diagnostic criteria/guidelines (please specify)
  - ☐ No
  - ☐ Yes, specify …..

1.6 indicate the roles of who should manage FM patients?

- Orthopedic doctor
  - ☐ Primary role
  - ☐ Secondary role
  - ☐ No role
  - ☐ I don't know

- Rheumatologist doctor
  - ☐ Primary role
  - ☐ Secondary role
  - ☐ No role
  - ☐ I don't know

- Psychiatrist doctor
  - ☐ Primary role
  - ☐ Secondary role
  - ☐ No role
  - ☐ I don't know

- Neurologist doctor
  - ☐ Primary role
  - ☐ Secondary role
  - ☐ No role
  - ☐ I don't know

- Pain management doctor
  - ☐ Primary role
  - ☐ Secondary role

☐ No role
☐ I don’t know

- General practitioner doctor
  ☐ Primary role
  ☐ Secondary role
  ☐ No role
  ☐ I don’t know

- Physical therapist
  ☐ Primary role
  ☐ Secondary role
  ☐ No role
  ☐ I don’t know

- Psychologist
  ☐ Primary role
  ☐ Secondary role
  ☐ No role
  ☐ I don’t know

- Multidisciplinary team
  ☐ Primary role
  ☐ Secondary role
  ☐ No role
  ☐ I don’t know

- Other profession (please specify …..)

- 4.7 indicate whether or not each of the following is a risk factor for developing FM?

• Genetic
  ☐ Risk factor for FM
  ☐ Not a risk factor for FM
  ☐ I don’t know

• Poor sleep
  ☐ Risk factor for FM
  ☐ Not a risk factor for FM
  ☐ I don’t know

• Obesity
  ☐ Risk factor for FM
  ☐ Not a risk factor for FM
  ☐ I don’t know

• Physical inactivity

☐ Risk factor for FM
☐ Not a risk factor for FM
☐ I don’t know

- Poor mental health
  ☐ Risk factor for FM
  ☐ Not a risk factor for FM
  ☐ I don’t know

- Stress
  ☐ Risk factor for FM
  ☐ Not a risk factor for FM
  ☐ I don’t know

- Gender
  ☐ Risk factor for FM
  ☐ Not a risk factor for FM
  ☐ I don’t know

- Age
  ☐ Risk factor for FM
  ☐ Not a risk factor for FM
  ☐ I don’t know
- Other risk factors for FM please specify …

- 4.8 Please indicate the degree of recommendation of the following in the management of patients with FM? (Select one or more from the following) *
  - Pain education
    ☐ Highly recommended
    ☐ Recommended
    ☐ Neutral
    ☐ Not recommended
    ☐ I don’t know

  - Self-management
    ☐ Highly recommended
    ☐ Recommended
    ☐ Neutral
    ☐ Not recommended
    ☐ I don’t know

  - Non-narcotics analgesics
    ☐ Highly recommended
    ☐ Recommended
    ☐ Neutral
    ☐ Not recommended
    ☐ I don’t know

- Narcotics medication (opioids)
  - ☐ Highly recommended
  - ☐ Recommended
  - ☐ Neutral
  - ☐ Not recommended
  - ☐ I don't know

- Antidepressant drugs
  - ☐ Highly recommended
  - ☐ Recommended
  - ☐ Neutral
  - ☐ Not recommended
  - ☐ I don't know

- Muscle relaxant drugs
  - ☐ Highly recommended
  - ☐ Recommended
  - ☐ Neutral
  - ☐ Not recommended
  - ☐ I don't know

- Aerobic exercises
  - ☐ Highly recommended
  - ☐ Recommended
  - ☐ Neutral
  - ☐ Not recommended
  - ☐ I don't know

- Resistance exercises
  - ☐ Highly recommended
  - ☐ Recommended
  - ☐ Neutral
  - ☐ Not recommended
  - ☐ I don't know

- Cognitive behavioral therapy (CPT)
  - ☐ Highly recommended
  - ☐ Recommended
  - ☐ Neutral
  - ☐ Not recommended
  - ☐ I don't know

- Manual therapy
  - ☐ Highly recommended
  - ☐ Recommended
  - ☐ Neutral

☐ Not recommended
☐ I don’t know

- Dry needling
  ☐ Highly recommended
  ☐ Recommended
  ☐ Neutral
  ☐ Not recommended
  ☐ I don’t know

- Ultrasound/electrotherapy
  ☐ Highly recommended
  ☐ Recommended
  ☐ Neutral
  ☐ Not recommended
  ☐ I don’t know

- Massage
  ☐ Highly recommended
  ☐ Recommended
  ☐ Neutral
  ☐ Not recommended
  ☐ I don’t know

- Trigger point release
  ☐ Highly recommended
  ☐ Recommended
  ☐ Neutral
  ☐ Not recommended
  ☐ I don’t know

- Acupuncture
  ☐ Highly recommended
  ☐ Recommended
  ☐ Neutral
  ☐ Not recommended
  ☐ I don’t know

- Diet
  ☐ Highly recommended
  ☐ Recommended
  ☐ Neutral
  ☐ Not recommended
  ☐ I don’t know

- Sleep hygiene

☐ Highly recommended
☐ Recommended
☐ Neutral
☐ Not recommended
☐ I don’t know

- 4.9 Please indicate whether each of the following statements are true or false.
  - Fibromyalgia is characterized by widespread musculoskeletal pain
    ☐ True
    ☐ False
    ☐ I don’t know

  - Fibromyalgia is an inflammatory joint disease
    ☐ True
    ☐ False
    ☐ I don’t know

  - Fibromyalgia is an objectively defined condition (i.e., not a diagnosis of exclusion)
    ☐ True
    ☐ False
    ☐ I don’t know

  - Fibromyalgia is equally seen in females and males
    ☐ True
    ☐ False
    ☐ I don’t know

  - Fibromyalgia causes deformity in joints
    ☐ True
    ☐ False
    ☐ I don’t know

  - Fibromyalgia may cause forgetfulness and concentration difficulties
    ☐ True
    ☐ False
    ☐ I don’t know

  - Fibromyalgia is a disease that can be healed completely
    ☐ True
    ☐ False
    ☐ I don’t know

  - Sleep disturbance is common in fibromyalgia
    ☐ True
    ☐ False
    ☐ I don’t know

- Fibromyalgia is completely psychological
  - ☐ True
  - ☐ False
  - ☐ I don't know

- Fibromyalgia affects the quality of life
  - ☐ True
  - ☐ False
  - ☐ I don't know

- Fibromyalgia treatment depend mainly in medications
  - ☐ True
  - ☐ False
  - ☐ I don't know

- Lab test (e.g. muscle biopsy) and imaging are helpful to confirm the diagnosis of fibromyalgia
  - ☐ True
  - ☐ False
  - ☐ I don't know

- 11 out of 18 tender points are important in the diagnosis of fibromyalgia
  - ☐ True
  - ☐ False
  - ☐ I don't know

- Headache is useful in the diagnosis of fibromyalgia
  - ☐ True
  - ☐ False
  - ☐ I don't know

- Fatigue is useful in the diagnosis of fibromyalgia
  - ☐ True
  - ☐ False
  - ☐ I don't know

- Anxiety and/or depression are useful in the diagnosis of fibromyalgia
  - ☐ True
  - ☐ False
  - ☐ I don't know

- Waking up tired (unrefreshed) is useful in the diagnosis of fibromyalgia
  - ☐ True
  - ☐ False
  - ☐ I don't know

Thank you for taking the time to complete the survey
